# Opportunities and Challenges Addressing Access to Healthy Food in Five Rural Louisiana Food Stores

**DOI:** 10.5888/pcd16.190118

**Published:** 2019-07-18

**Authors:** Michelle Kendall, Stephanie T. Broyles, Jamila Freightman, Melissa Cater, Denise Holston

**Affiliations:** 1 Pennington Biomedical Research Center, Baton Rouge, Louisiana; 2 Louisiana State University Health Sciences Center, School of Public Health, New Orleans, Louisiana; 3 Louisiana State University Agricultural Center, School of Nutrition and Food Science, Baton Rouge, Louisiana

## Abstract

The prevalence of high obesity in rural communities may result from low access to healthy foods. To improve the local food environment, a multicomponent environmental food store intervention was implemented in 3 Louisiana parishes where obesity prevalence was greater than 40%. The intervention consisted of healthy-food demonstrations, in-store marketing, and encouraging store owners to stock healthy items. We documented aspects of the rural food store climate, such as store size and the store owner’s willingness to stock healthy items, that affect improving access to healthy food. We found that although the intervention was not effective in shifting purchasing or dietary habits of customers, positive changes in some food store environments did occur. To maximize the effect that rural food store interventions can have on reducing obesity, it is essential to understand aspects of the rural food store climate.

**Summary**

**What is already known about this topic?**

Environmental food store interventions are recommended to develop healthy food environments and to reduce obesity.

**What is added by this report?**

A multicomponent environmental food store intervention was implemented in 5 rural food stores across 3 Louisiana parishes with high obesity prevalence to address healthy food access.

**What are the implications for public health practice?**

We highlight the successes and challenges of working in the rural food store climate as well as results of our program evaluation on health behavior change. By understanding the rural food store climate, public health practitioners can tailor best practices to reduce obesity for rural populations.

## Background

Louisiana consistently ranks in the top 10 states with the highest prevalence of obesity, and in 2017, 36.2% of Louisiana adults were obese (1). To combat rising obesity, Louisiana State University’s (LSU’s) AgCenter’s Cooperative Extension Healthy Communities initiative created cross-sector partnerships with schools, elected officials, community members, faith-based communities, and community stakeholders to promote healthy eating and physical activity through policy, systems, and environmental approaches. The Healthy Communities initiative began in 2015 and initially targeted 3 rural Louisiana parishes (counties): Madison (population, 11,616; adult obesity prevalence, 43.4%); St. Helena (population, 10,509; adult obesity prevalence, 41.9%); and Tensas (population, 4,771; adult obesity prevalence, 41.8%) (2,3). A central component of this initiative was the development of local Healthy Communities coalitions that assess local needs and prioritize interventions targeting the local nutrition and physical activity environments.

In response to coalition feedback, the Healthy Communities initiative implemented multipronged interventions in 5 food stores in the 3 parishes in fall 2017. The interventions aimed to increase the community’s awareness of healthy food offerings and to increase access to healthy foods and included the following components: healthy food demonstrations, in-store marketing, and encouraging store owners to stock healthy items.

## Intervention Approach

Healthy Communities Coalition members and program staff members worked through a collaborative needs assessment process to identify local resources, including existing food stores. In some cases, coalition members introduced LSU AgCenter staff to local food store owners. If contact was more difficult to make, the program staff conducted outreach to food stores and invited store owners to attend Healthy Communities Coalition meetings. In one Healthy Communities Coalition, the store owner regularly attended coalition meetings. Owners from at least one major food store, grocery, or convenience store were recruited in each parish. To improve the intervention’s reach, our approach was geared toward engaging owners of local stores recommended by community members as being locations where they frequently shopped. All store owners who were invited to participate agreed to do so. In total, 5 stores (3 grocery stores and 2 convenience stores) across the 3 parishes became Healthy Communities Partner Stores.

In June 2017, store owners received technical assistance through in-person site visits regarding strategies to promote healthy food items from a consultant with The Food Trust, a national healthy food access organization. Store owners were asked to provide input on available marketing materials that worked best for their store, were given shelving and cooler infrastructure, and asked about aesthetic preferences. Store owners selected the most applicable and feasible interventions to improve healthy food access and awareness in their store. All stores chose to participate in in-store marketing and nutrition education, and LSU AgCenter program staff members led implementation to minimize the burden on the food store staff. Although no monetary incentives were provided, store owners did receive marketing materials at no cost. The marketing materials were provided by the LSU AgCenter and valued at $1,000 per store. Store owners were also encouraged to stock healthy items; however, this was voluntary. One store used a merchandising store “reset” (large scale rearrangement of a store’s products) as an opportunity to integrate healthy food products into their store.

In-store marketing included shelf banners and signage that used a traffic-light concept to help customers identify healthy and unhealthy options. Green signals “Go,” indicating the healthiest foods; yellow signals “Caution,” indicating somewhat healthy foods; and red signals “Stop and Think,” indicating the least healthy foods (Table 1). Marketing was installed throughout partner stores in produce, dairy, and meat departments and on aisles of canned and frozen goods, bread, pasta, and cereal. Grab-and-go coolers with beverages and snack items were also targeted. In-store marketing was installed in all partner stores over a 4-month period (September–December 2017). The in-store marketing exposure period ranged from 8 to 12 months (August 2017–August 2018). LSU AgCenter staff members conducted in-store nutrition education lessons, including food demonstrations and healthy food taste tests, on at least a quarterly basis during the intervention period.

**Table 1:** In-store Marketing Using Traffic Light Concept to Indicate Healthy Foods, Louisiana 2017–2018

	Green – Go: Healthiest	Yellow – Caution: Somewhat Healthy	Red – Stop: Least Healthy
Fruits and vegetables	Fresh fruits and vegetables	Canned or frozen fruits and vegetables with less than 290 mg sodium and no added sugar	Canned or frozen fruits and vegetables with more than 290 mg sodium or with added sugar
Grains	Whole grains listed as the first ingredient: pasta, rice, bread, flour	Refined and whole grain: whole grain is not listed as first ingredient	White refined: whole grain not listed as an ingredient
Proteins	Lean and low-fat fish, poultry, eggs, beef, pork	Non-lean meat: steak, ground beef, poultry with skin	Processed meats: high sodium or high fat meats – bacon, deli meat, sausage
Beverages	No sugar added, water, fat-free, or 1% low-fat milk	100% juice, diet drinks, low-fat chocolate milk	Soda, fruit drinks, sport drinks, iced tea, lemonade

## Implications for Public Health

Several aspects of the rural food store climate emerged as important considerations when implementing environmental food store interventions in rural areas. First, the size of the store and its ownership dictates the store’s ability to stock healthy items. Four of 5 partner stores were independently owned and operated by people residing in the parish their store served. One store was owned by a local grocery retail chain. All 3 grocery stores ordered and received products through large, full-service warehouse distribution centers. Stores are required to sign contracts for numerous years and must attain certain sales levels to meet contract requirements. Therefore, larger stores had better access to new, healthy products and an easier time sourcing them. These stores also received additional assistance in many areas such as payroll, transaction processing equipment, bookkeeping, and store merchandising resets.

Smaller stores, such as the convenience stores we worked with, did not have these amenities and therefore had a limited capacity and ability to stock healthy items. One store owner commented that his store had been on decline since the 1970s as families continued to move out of the parish, resulting in reduced sales and a store with smaller capacity. As stores get smaller, they no longer meet wholesaler contracting requirements. Without these contracts, small store owners must source independent vendors to stock their stores. However, given the rural location of stores, they encounter difficulties in procuring vendors that are willing to make long-distance deliveries, particularly fresh-produce vendors. One store owner mentioned that during spring and summer months, when local community members have gardens, he buys produce from gardeners to supplement his produce department. Therefore, less traditional routes of procuring healthy foods, such as working with seasonal local gardeners, may be an opportunity to explore in rural areas as this work continues. These store owners also mentioned that they were less willing to order new products for their stores because they felt there was a high chance the products would not sell. In these cases, we focused on promoting the healthy items that already existed, thereby increasing awareness of existing healthy foods as opposed to increasing access to additional healthy foods.

A second aspect of the food store climate relates to the importance of food and beverage companies. Across all stores, many point-of-purchase areas (strategically placed displays or coolers that aim to attract customers) could not be altered or changed because of contracts in place with large food and beverage companies, such as Coca Cola, Little Debbie, Pepsi, and Frito-Lay. These companies supply infrastructure (shelving or coolers) for products and have local company representatives stock products weekly, reducing burden on the store’s staff. Items with high sugar and sodium content were available at point-of-purchase areas, including the checkout aisle. Our experiences are echoed in a recent study of agreements between food stores and food and beverage distributors (4). These distributors influence what foods are stocked in stores, and in turn, what foods are available for customers to purchase.

Despite difficulties in accessing and sourcing new, healthy products, we saw increases in healthy food offerings in partner stores overall (Table 2). Two store owners voluntarily increased available healthy items. These increases were due in part to larger (grocery) stores being able to stock new items through merchandising store resets and using such resets as an opportunity to stock healthy products. One of these stores also implemented a healthy checkout aisle stocked with healthy grab-and-go snacks strategically placed at the point of checkout. Pre-intervention and post-intervention store inventories showed that in-store healthy food availability increased the most for canned fruits and vegetables and whole-grain cereal. These positive findings are supported by a previous study of rural food store owners indicating that owners are willing to stock healthy items (5).

**Table 2:** Baseline and Post-Intervention Availability of Healthy Food Offerings^a^ Across 5 Healthy Community Partner Stores, Louisiana 2017–2018

Food	Baseline	Post-Intervention
Fresh fruit	27	25
Fresh vegetables	40	45
Canned fruit	16	22
Canned vegetables	39	64
Frozen fruit or vegetables	70	68
Skim or low-fat milk	8	3
Whole grain bread	3	5
Whole grain cereal	9	24
Lean cuts of meat	1	1
Dried beans or peas	14	18

a Average number of varieties.

Future interventions should carefully consider whether the intervention strength (eg, dose, reach) is adequate to promote behavior change. In our study, 63% of customers said that they noticed signage for healthy foods and drinks in the partner store before signage was installed; these results may indicate that customers may not have noticed the implementation of in-store marketing or that survey responses were subject to social desirability bias. A similar study assessing customer reactions to healthy in-store marketing interventions found that few customers noticed program interventions, which included in-store marketing, and noted that more marketing promotion was needed (6). Stronger cross-promotion or reinforcement of marketing with nutrition education lessons (eg, food demonstrations) or additional strategies, such as in-store advertisements or loud speaker announcements, may be necessary to increase customer exposure to marketing through direct customer contact. Furthermore, pre–post assessments (52 customer surveys pre-intervention and 78 surveys 8 to 11 months post-intervention) revealed no changes in customer perceptions about the local food environment or self-reported purchase and consumption of healthy (eg, fruits, vegetables) and unhealthy (eg, soda) foods. At both time points, 40% of customers at baseline and 38% post-intervention reported purchasing fruits or vegetables from the partner store at least once in the past week. It is possible that the level of in-store marketing and nutrition education as implemented was not a sufficient dose to produce the desired behavior changes. Previous food store interventions that were successful at producing purchasing or dietary changes had at least medium to high dose (exposure), reach (number of participants reached), and fidelity (program implemented as planned), and achieved dose and reach through multipronged strategies (7) that combined behavioral and environmental approaches.

Interestingly, customers surveyed at the partner food stores reported positive perceptions of their local food environment, despite living in rural food deserts (rural areas more than 10 miles from a grocery store or supermarket) (8). A recent study in a rural agricultural community found that community members felt that they had adequate access to healthy foods and perceived a positive food environment whereas the objective measurement of their local food environment indicated lack of access to healthy foods, a poor food environment (9). Therefore, individual perceptions of the local food environment may not be helpful in indicating the success of interventions aimed at increasing healthy food access and awareness.

Currently, interventions, including enhanced in-store marketing, that engage local food retailers are promoted as best practices to encourage the development of healthy food environments and to reduce obesity (10). Our assessment further identifies rural food store owners as important stakeholders in addressing rural healthy food access. Although we documented successes in large food stores, aspects of the rural food store climate require consideration for feasible approaches in these small stores, given the limitations of smaller stores’ ability to source a wide variety of healthy foods. Our assessment highlights important aspects to inform ongoing efforts addressing rural healthy food access.
